# Cimicifugae Rhizoma Extract Attenuates Oxidative Stress and Airway Inflammation via the Upregulation of Nrf2/HO-1/NQO1 and Downregulation of NF-κB Phosphorylation in Ovalbumin-Induced Asthma

**DOI:** 10.3390/antiox10101626

**Published:** 2021-10-15

**Authors:** Je-Oh Lim, Kwang Hoon Song, Ik Soo Lee, Se-Jin Lee, Woong-Il Kim, So-Won Pak, In-Sik Shin, Taesoo Kim

**Affiliations:** 1 College of Veterinary Medicine and BK21 FOUR Program, Chonnam National University, 77 Yongbong-ro, Buk-gu, Gwangju 61186, Korea; 166634@jnu.ac.kr (J.-O.L.); 196433@jnu.ac.kr (S.-J.L.); 208521@jnu.ac.kr (W.-I.K.); 208514@jnu.ac.kr (S.-W.P.); 2 KM Convergence Research Division, Korea Institute of Oriental Medicine, 1672 Yuseongdae-ro, Yuseong-gu, Daejeon 34054, Korea; ksong@kiom.re.kr (K.H.S.); knifer48@kiom.re.kr (I.S.L.); 3 R&D Strategy Division, Korea Institute of Oriental Medicine, 1672 Yuseongdae-ro, Yuseong-gu, Daejeon 34054, Korea

**Keywords:** Cimicifugae Rhizoma, asthma, nuclear factor erythroid 2-related factor 2 (Nrf2), heme oxygenase-1 (HO-1), nuclear factor-κB (NF-κB), matrix metalloproteinase-9 (MMP-9)

## Abstract

Cimicifugae Rhizoma has been used as a medicinal herb for fever, pain, and inflammation in East Asia. We conducted this study because the effect of Cimicifugae Rhizoma extract (CRE) on allergic asthma has not yet been evaluated. To induce allergic airway inflammation, we intraperitoneally injected ovalbumin (OVA) mixed with aluminum hydroxide into mice twice at intervals of 2 weeks (Days 0 and 14) and then inhaled them thrice with 1% OVA solution using a nebulizer (Days 21 to 23). CRE (30 and 100 mg/kg) was administered orally daily for 6 days (Days 18 to 23). The mice showed remarkable reduction in allergic inflammation at 100 mg/kg of CRE, as evidenced by decreased inflammatory cell counts, pro-inflammatory cytokine levels, OVA-specific immunoglobulin E level, airway hyperresponsiveness, and production of mucus. Additionally, these effects were involved with the enhancement of heme oxygenase-1 (HO-1), NAD(P)H: quinone oxidoreductase (NQO1), and nuclear factor erythroid 2-related factor 2 (Nrf2) expression and reduction of nuclear factor-κB (NF-κB) phosphorylation and matrix metalloproteinase-9 expression. Our findings indicated that CRE effectively protected against OVA-induced inflammation and oxidative stress via upregulation of the Nrf2/HO-1/NQO1 signaling and downregulation of NF-κB phosphorylation in asthma caused by OVA.

## 1. Introduction

Allergic asthma is a complex inflammatory disease of the lungs that affects 1 in 10 children and 1 in 12 adults globally. It is characterized by airway hyperresponsiveness (AHR), eosinophilic inflammation, and mucus overproduction [1]. Exposure to specific allergens triggers a type 2 immune response that is mediated by inflammatory cytokines that are involved with the production of immunoglobulin (Ig) E and eosinophilia [2]. Though several studies have been conducted to develop drugs that treat allergic asthma, there is still a lack of therapeutic agents that show no adverse effects and excellent therapeutic effects.

Oxidative stress is a condition where an imbalance between oxidants and antioxidants may cause biological damage to organisms [3]. Reactive oxygen species (ROS) are excessively produced in asthma, causing a decline in the antioxidant system function and an increase in the production of inflammatory mediators [4]. In particular, the decrease in heme oxygenase-1 (HO-1), NAD(P)H: quinone oxidoreductase (NQO1), and nuclear factor erythroid 2-related factor 2 (Nrf2), which is associated with the activation of the antioxidant system, is characteristically observed [5]. Excessive oxidative stress induces the activation of nuclear factor-κB (NF-κB), which eventually causes matrix metalloproteinase-9 (MMP-9) overexpression and results in airway remodeling [6,7]. MMP-9 also aggravates airway inflammatory responses as it induces the elevation of inflammatory cytokines and chemokines [7].

Cimicifugae Rhizoma is a traditional herbal medicine that has been taken as an analgesic, anti-inflammatory, antipyretic, and antiviral in East Asia [8,9,10,11]. It demonstrated an antioxidant effect via its metal-chelating and radical-scavenging activities in in vitro experiments [12]. In addition, Cimicifugae Rhizoma ameliorates Poly (I: C)-induced airway inflammation, suggesting that Cimicifugae Rhizoma could be applied to treating various respiratory diseases such as asthma and chronic obstructive pulmonary disease [13]. Based on previous studies about the anti-inflammatory and antioxidant properties of Cimicifugae Rhizoma, we considered that Cimicifugae Rhizoma may effectively suppress asthmatic responses including airway inflammation, airway hyperresponsiveness (AHR), and mucus secretion in an experimental asthma model. However, no studies related to the therapeutic effect of Cimicifugae Rhizoma on allergic asthma have been reported so far.

Therefore, we investigated the therapeutic effects of Cimicifugae Rhizoma on ovalbumin (OVA)-induced asthma and analyzed the expression of proteins including Nrf2, HO-1, NQO1, MMP-9, and NF-κB, focusing on antioxidant functions, to elucidate the mechanism of action of Cimicifugae Rhizoma in order to prove its potential as an asthma treatment.

## 2. Materials and Methods

### 2.1. Plant Material

Cimicifugae Rhizoma was obtained from herb good manufacturing practice (hGMP) manufacturer (Omniherb, Seoul, Korea) in September 2020 and identified by an herbarium botanist. A voucher specimen (#KJE-74) of raw materials has been deposited in the Herbarium of Korea Institute of Oriental Medicine (KIOM, Daejeon, Korea).

### 2.2. Chemicals and Reagents

Caffeic acid, ferulic acid, isoferulic acid, cimicifugic acid B, cimicifugic acid F, OVA, and aluminum hydroxide were obtained from Sigma-Aldrich (St. Louis, MO, USA). The purity of all reference standards was >99%. High-performance liquid chromatography (HPLC) grade Acetonitrile, methanol, and water were obtained from J.T. Baker (Phillipsburg, NJ, USA). Formic acid was obtained from Merck (Darmstadt, Germany). Enzyme-linked immunosorbent assay (ELISA) kits for IL-4, IL-5, and IL-13 were obtained from R&D Systems (Minneapolis, MN, USA), and OVA-specific IgE was obtained from BioLegend Inc. (San Diego, CA, USA). Bradford assay reagent (Bio-Rad Laboratories, Hercules, CA, USA) was used. The antibodies, including β-actin, Nrf2, HO-1, NQO1, GSH, MMP-9, and NF-κB, were obtained from Abcam (Cambridge, MA, USA) or Cell Signaling (Boston, MA, USA).

### 2.3. Preparation of Sample and Standard Solution

Cimicifugae Rhizoma (1 kg) was extracted using 70% ethanol (10 L) at 80 °C for 3 h using a heat-reflux extractor. The extracted solution was filtered by a standard stainless sieve No. 270 (53 µm, ChunggyeSieve, Seoul, Korea) and evaporated under reduced pressure using a rotary evaporator (N-1200A, Eyela, Tokyo, Japan) at 50 °C and then freeze dried using a freeze dryer (FDU-2100, Eyela, Tokyo, Japan) at −80 °C for 72 h to obtain the extract powder (114.9 g, Yield 11.5%). This Cimicifugae Rhizoma extract (CRE, 100 mg) dissolved in methanol (10 mL) was filtered through a syringe filter (0.45 μm, Whatman, Clifton, NJ, USA) prior to injection. Standard stock solutions of 5 reference standards (all at 1 mg/mL) were prepared in methanol, stored at <4 °C, and used for HPLC analyses after serial dilution in methanol.

### 2.4. HPLC Condition

HPLC analyses were conducted using an Agilent 1200 HPLC instrument (Agilent Technologies, Santa Clara, CA, USA) equipped with an auto-sampler (G1329A), binary pump (G1312A), column compartment (G1316A), diode array detector (DAD, 1365B), and vacuum degasser (G1322A), and data were collected and analyzed using Agilent ChemStation software. Chromatographic separation was conducted using a Zorbax Eclipse Plus (250 mm × 4.6 mm, 5.0 μm; Agilent) and the column temperature was maintained at 40 °C. HPLC analyses were performed according to the previously described procedure with slight modification [14]. The mobile phase consisted of 0.1% formic acid in water (A) and acetonitrile (B) with gradient elution for better separation. The gradient solvent system was optimized as follows: 90–65% A (0–50 min), 65–0% A (50–55 min), 100% B (55–65 min), and 90% A (65–75 min) at a flow rate of 1.0 mL/min. The detection was conducted at 320 nm and the injection volume of each sample was 5 μL. To test for linearity, standard solutions were prepared at 5 levels by serially diluting the stock solution. Each analysis was repeated thrice, and the calibration curves were fitted using linear regression. The limit of detection (LOD) and limit of quantification (LOQ) data obtained under the optimal chromatographic conditions were determined using signal-to-noise (S/N) ratios of 3 and 10, respectively.

### 2.5. Experimental Animals and Protocol

Female 6-week-old specific pathogen-free BALB/c mice (SAMTAKO, Osan, Korea) were housed under standard conditions (12 h night/12 h day cycle, humidity 55 ± 5%, temperature 22 ± 2 °C) and were provided ad libitum access to food and water. All the procedures of animal experimentation were approved by the Institutional Animal Care and Use Committee of Chonnam National University (CNU IACUC-YB-2020-99).

Animals were randomly assigned into 5 groups (*n* = 5–7) after a week of acclimation: normal control (NC), OVA, dexamethasone (DEX, 2 mg/kg), CRE30 (30 mg/kg), and CRE100 (100 mg/kg). All animals except the NC group were intraperitoneally administered with OVA (20 µg/mouse) dissolved in aluminum hydroxide (2 mg/mouse) on days 0 and 14 and then inhaled with OVA solution (1%) for 1 h by a nebulizer (Omron, Tokyo, Japan) on days 21 to 23. Cimicifugae Rhizoma extract (CRE) and dexamethasone (DEX) were administered via oral gavage once a day from days 18 to 23. On day 24, AHR was determined after methylcholine inhalation (0, 5, 10, and 15 mg/mL) for 3 min using a whole-body plethysmograph (Allmedicus, Seoul, Korea). The data were presented as the dimensionless parameter enhanced pause (Penh).

### 2.6. Bronchoalveolar Lavage Fluid (BALF) and Serum Analysis

On day 25, we conducted tracheostomy to obtain BALF in mice under anesthesia. After endotracheal tubes were inserted into the incised trachea, the lungs were lavaged twice with ice-cold PBS (0.7 mL) and the obtained BALF samples were centrifuged. The inflammatory cytokines in the supernatant were measured using ELISA and inflammatory cell count in the BALF pellets was measured using a Countess II Cell Counter (Thermo Fisher Scientific, San Diego, CA, USA). Differential cell counts of BALF ware performed as previously described [15]. The collected blood samples from cauda vena cava were centrifuged to obtain serum for analyzing OVA-specific IgE using the ELISA kit.

### 2.7. Histological Examination of Lung Tissue

The right lung was resected, fixed using 10% neutral buffered formalin, embedded in paraffin, and cut into 4 µm thick sections. To determine inflammation and mucus secretion, the tissues were stained with hematoxylin and eosin (Sigma-Aldrich, St. Louis, MO, USA), and periodic acid–Schiff (Abcam, Cambridge, UK), respectively. Quantification of inflammation and mucus secretion were determined by an image analyzer (IMT i-Solution Inc., Vancouver, BC, Canada). Immunohistochemical analysis was conducted using the anti-mouse HO-1 antibody (diluted 1:200) and a commercial kit (Vector Laboratories, Burlingame, CA, USA) using the method described earlier [15] to evaluate HO-1 expression. In addition, we carried out immunofluorescence analysis to assess MMP-9 expression using anti-mouse MMP-9 antibody (diluted 1:200). The slides were mounted using Prolong Gold antifade with DAPI (Thermo Fisher Scientific, San Diego, CA, USA) and evaluated by confocal microscopy (LSM980, Carl Zeiss, Oberkochen, Germany).

### 2.8. Western Blot

The lung tissues were minced and homogenized, followed by centrifugation at 14,000 rpm (10 min, 4 °C). Protein concentration was evaluated using the Bradford reagent. Each sample (30 μg protein) was separated using 10% SDS-polyacrylamide gel electrophoresis (PAGE) and transferred to 0.25 µm PVDF membranes (Millipore, Burlington, MA, USA). The membranes were then blocked with 5% skimmed milk at room temperature for 2 h, incubated with primary antibodies (1:1000) overnight at 4 °C, then incubated with horseradish peroxidase-conjugated secondary antibody (1:10,000) for 2 h, and finally developed using an enhanced chemiluminescence kit (Thermo Fisher Scientific, San Diego, CA, USA). The densitometric value of protein band was determined by Chemi-Doc (Bio-Rad Laboratories, Hercules, CA, USA).

### 2.9. Gelatin Zymography

To measure the activity of MMP-9, equal amounts of lung proteins, extracted in the same way as mentioned above, were loaded for gelatin zymography (30 μg/lane). In brief, gels were prepared using 10% SDS-PAGE containing 1% gelatin as a substrate for MMPs. After washing with 2.5% Triton X-100 (Sigma-Aldrich, St. Louis, MO, USA) for 45 min to remove SDS, the gels were incubated at 37 °C for 16 h in a developing buffer containing 1 M Tris-HCl and 10 mM CaCl_2_. Subsequently, Coomassie brilliant blue stain (Daejung, Siheung, Korea) was applied on the gels. Gelatinase activity appeared as white bands on a blue background.

### 2.10. Statistical Analysis

The data are represented as the mean ± standard deviation (SD). Statistical significance was evaluated using analysis of variance followed by a multiple comparison test with Dunnett’s adjustment. Statistical significance was considered at *p* < 0.05.

## 3. Results

### 3.1. HPLC Analysis of CRE

**Figure 1:**
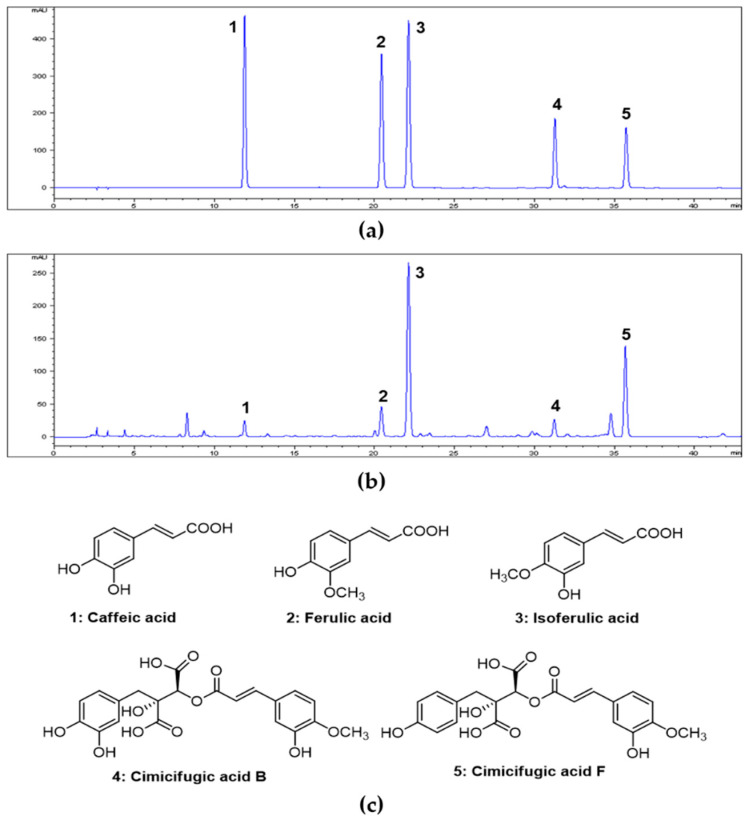
High-performance liquid chromatography (HPLC) chromatograms of five standard compound mixtures (**a**) and 70% ethanol extract of Cimicifugae Rhizoma (**b**). Chemical structures of five compounds (**c**). Peak identification: **1**, caffeic acid; **2**, ferulic acid; **3**, isoferulic acid; **4**, cimicifugic acid B; **5**, cimicifugic acid F. Chromatographic conditions are described in the text. Detection was at 320 nm.

HPLC analysis of the 70% ethanol CRE showed the presence of two major peaks (peaks 3 and 5) in addition to several minor peaks (Figure 1a). By comparing retention times and UV spectra of these peaks with those of the corresponding standards (Figure 1b), the compounds corresponding to peaks 1–5 were identified as caffeic acid, ferulic acid, isoferulic acid, cimicifugic acid B, and cimicifugic acid F (Figure 1c), respectively. Detailed information regarding the calibration curve, linear range, LOD, and LOQ for the five reference standards is listed in Supplementary Materials Table S1. Each calibration curve possessed good linearity (R^2^ > 0.999) within the selected range. The developed HPLC method was applied to simultaneously determine 5 compounds in the CRE. The contents of the five compounds (peaks 1–5) found in the CRE were 0.06%, 0.14%, 0.81%, 0.19%, and 1.09%, respectively (Table 1).

### 3.2. CRE Inhibited Eosinophilia and Airway Hyperresponsiveness (AHR) in Animals with Asthma

**Figure 2:**
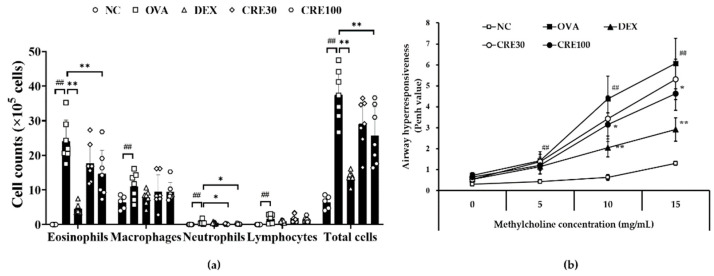
CRE inhibited inflammatory cell count and airway hyperresponsiveness (AHR) in animals with asthma. (**a**) Inflammatory cell counts in BALF, (**b**) AHR. NC: PBS treatment and PBS challenge; OVA: PBS treatment and OVA challenge; DEX: dexamethasone (2 mg/kg) treatment and OVA challenge; CRE30 and CRE100: CRE (30 mg/kg and 100 mg/kg, respectively) and OVA challenge. The values are shown as the mean ± SD (*n* = 5–7). ^##^ *p* < 0.01 versus NC; *, ** *p* < 0.05 and 0.01 versus OVA.

The number of inflammatory cells in the BALF of the OVA group was higher than that in the NC group (Figure 2a). CRE groups showed a decrease in the number of inflammatory cells, particularly eosinophils, as compared to that in the OVA group, and there was a statistically marked difference with that of the CRE100 group.

AHR was noticeably elevated in the OVA group in comparison to the NC group (Figure 2b). However, CRE reduced AHR in animals with asthma by increasing the concentration of methylcholine. In particular, the CRE100 group revealed a marked decline in AHR as compared to the OVA group.

### 3.3. CRE Decreased Inflammatory Cytokines and OVA-Specific IgE in Animals with Asthma

**Figure 3:**
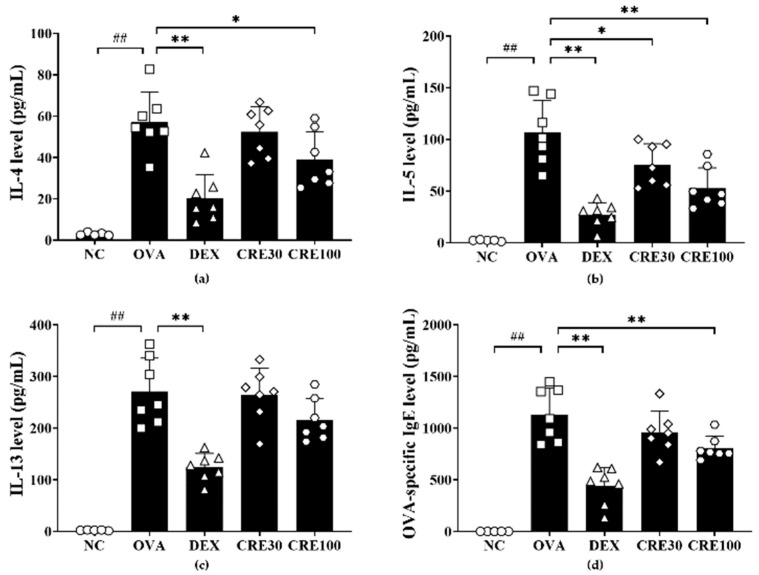
CRE decreased inflammatory cytokines and OVA-specific IgE in animals with asthma. (**a**) IL-4, (**b**) IL-5, (**c**) IL-13, (**d**) OVA-specific IgE. NC: PBS treatment and PBS challenge; OVA: PBS treatment and OVA challenge; DEX: dexamethasone (2 mg/kg) treatment and OVA challenge; CRE30 and CRE100: CRE (30 mg/kg and 100 mg/kg, respectively) and OVA challenge. The values are shown as the mean ± SD (*n* = 5–7). ^##^ *p* < 0.01 versus NC; *, ** *p* < 0.05 and 0.01 versus OVA.

The OVA group exhibited marked elevations in IL-4, -5, and -13 levels in BALF as comparison to those in the NC group (Figure 3a, Figure 3b and Figure 3c, respectively). By contrast, the CRE-treated groups demonstrated a decrease in inflammatory cytokine levels in the BALF of animals with asthma. In particular, IL-4 and -5 levels in the BALF from the high-dose CRE group displayed significant reductions in comparison with those in the OVA group. OVA-specific IgE was also found to be more elevated in the OVA group than in the NC group (Figure 3d). CRE100 group markedly decreased the OVA-specific IgE in comparison to the OVA group.

### 3.4. CRE Reduced Airway Inflammation and Mucus Secretion of Lung Tissue from Animals with Asthma

**Figure 4:**
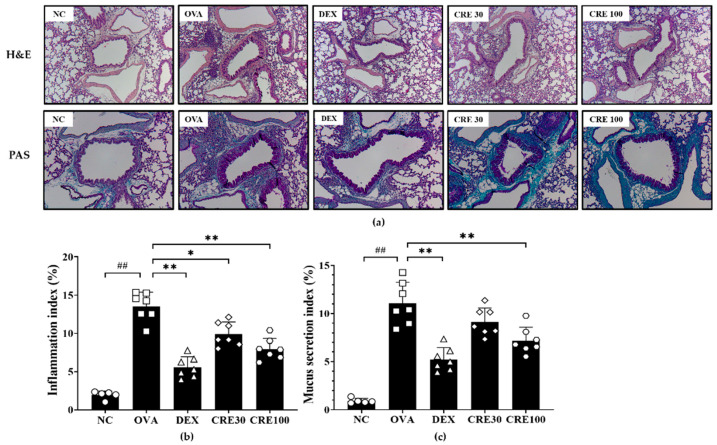
CRE attenuated airway inflammation and mucus secretion of lung tissue from animals with asthma. (**a**) Airway inflammation (H&E stain) and mucus secretion (PAS stain), (**b**) inflammatory index, (**c**) mucus secretion index. NC: PBS treatment and PBS challenge; OVA: PBS treatment and OVA challenge; DEX: dexamethasone (2 mg/kg) treatment and OVA challenge; CRE30 and CRE100: CRE (30 mg/kg and 100 mg/kg, respectively) and OVA challenge. The values are shown as the mean ± SD (*n* = 5). ^##^ *p* < 0.01 versus NC; *, ** *p* < 0.05 and 0.01 versus OVA.

The OVA group revealed noticeable inflammatory cell recruitment in the peribronchial and perivascular lesions as compared to that in the NC group (Figure 4a,b). By contrast, the high-dose CRE group exhibited a decrease in the inflammatory responses of lung tissue as compared to that in the OVA group. Mucus secretion was conspicuously increased in the OVA group as compared to that in the NC group, whereas it was declined in the CRE groups as compared to that in the OVA group (Figure 4a,c).

### 3.5. CRE Elevated the Expression of the Nrf2/HO-1/NQO1 in Animals with Asthma

**Figure 5:**
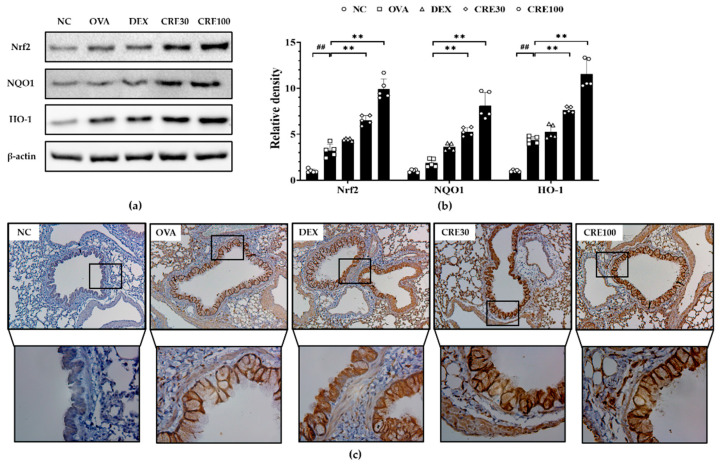
CRE increased the expression of Nrf2/HO-1/NQO1 in animals with asthma. (**a**) The expression of Nrf2/HO-1/NQO1, (**b**) relative expression, (**c**) immunohistochemistry for HO-1. The black box is enlarged and shown in the lower histological figure of (**c**). NC: PBS treatment and PBS challenge; OVA: PBS treatment and OVA challenge; DEX: dexamethasone (2 mg/kg) treatment and OVA challenge; CRE30 and CRE100: CRE (30 mg/kg and 100 mg/kg, respectively) and OVA challenge. The values are shown as the mean ± SD (*n* = 5). ^##^ *p* < 0.01 versus NC; ** *p* < 0.01 versus OVA.

The expression of Nrf2 and NQO1 was markedly decreased in the OVA group compared to that in the NC group (Figure 5a,b). By contrast, the CRE groups displayed significantly elevated expressions of Nrf2 and NQO1 as compared to that in the OVA group. In addition, HO-1 expression was markedly increased in the OVA group as compared to that in the NC group, whereas it was elevated in the CRE-treated groups as compared to that in the OVA group (Figure 5a–c).

### 3.6. CRE Suppressed NF-κB Phosphorylation and MMP-9 Expression in Animals with Asthma

**Figure 6:**
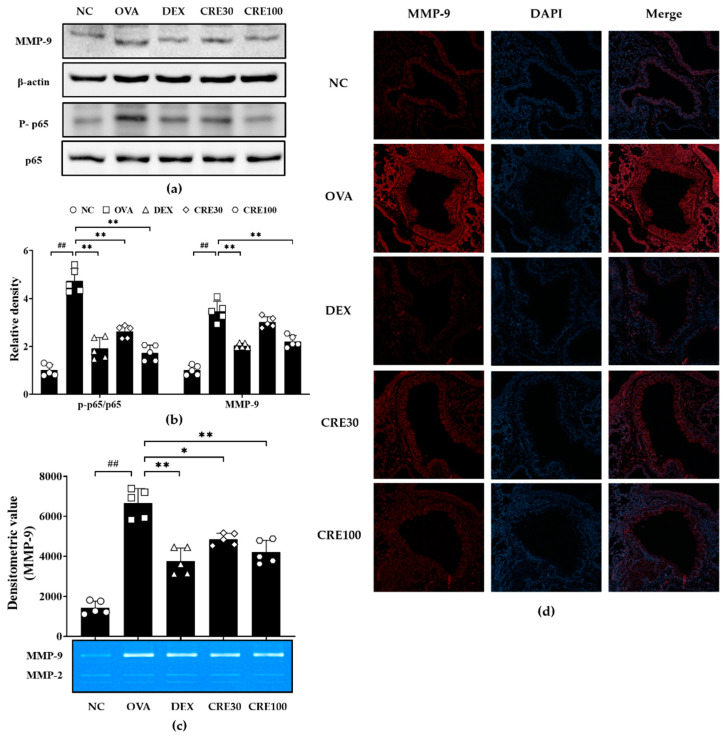
CRE suppressed NF-κB phosphorylation and MMP-9 expression in animals with asthma. (**a**) The expression of NF-κB and MMP-9, (**b**) relative density, (**c**) zymography for MMP-9, (**d**) immunofluorescence for MMP-9. NC: PBS treatment and PBS challenge; OVA: PBS treatment and OVA challenge; DEX: dexamethasone (2 mg/kg) treatment and OVA challenge; CRE30 and CRE100: CRE (30 mg/kg and 100 mg/kg, respectively) and OVA challenge. The values are shown as the mean ± SD (*n* = 5). ^##^ *p* < 0.01 versus NC; *, ** *p* < 0.05 and 0.01 versus OVA.

The OVA group revealed significantly elevated NF-κB phosphorylation in comparison with the NC group (Figure 6a,b). By contrast, the CRE groups displayed a noticeable reduction in phosphorylation of p65 as compared to that in the OVA group. The activity and expression of MMP-9 was markedly elevated in the OVA group as compared to that in the NC group (Figure 6a,c). By contrast, the CRE groups revealed a noticeable reduction in the expression and activity of MMP-9 as compared to that in the OVA group. Additionally, the reduction in MMP-9 induced by CRE treatment was also observed in histological examination. The OVA group revealed a noticeable elevation in MMP-9 expression in the lung tissue as compared to that in the NC group (Figure 6d). However, the CRE groups exhibited a significant decline in MMP-9 expression in the lung tissue as compared to that in the OVA group.

## 4. Discussion

Allergic asthma has been increasing every year, threatening human health. Various studies have been conducted to develop anti-asthmatic drugs [2,3]. Several researchers are developing anti-asthmatic drugs with a focus on anti-inflammatory and antioxidant materials because excessive inflammation and oxidative stress play a crucial role in the pathogenesis of asthma [1,3]. We investigated whether CRE has therapeutic effects on asthma induced by OVA. In addition, the expression of the proteins related to inflammatory and antioxidant systems was analyzed to clarify the mechanism of action of CRE. CRE treatment was found to inhibit asthmatic responses, including inflammatory cell accumulation, mucus overproduction, and increased AHR, with reductions in production of cytokines and OVA-specific IgE. Additionally, CRE activated Nrf2/HO-1/NQO1 signaling associated with the antioxidant system and suppressed NF-κB phosphorylation and expression of MMP-9, which are related to inflammatory reaction.

Eosinophilic inflammation of the lung is a prominent feature of asthma [16]. During the development of asthma, the interaction of antigen-presenting cells with sensitized helper T lymphocytes produces IL-4, IL-5, and IL-13, which influence the differentiation, survival, and function of eosinophils leading to inflammatory cell recruitment and mucus secretion with elevated AHR and allergen-specific IgE [17,18]. Thus, the reduction in cytokine levels is considered to be an important therapeutic strategy in the treatment of allergic asthma. CRE effectively inhibited the production of cytokines and OVA-specific IgE along with the noticeable decline in AHR and inflammatory cell accumulation. In addition, these effects of CRE were accompanied by histological changes in the lung tissue, such as the reduction in inflammatory cell infiltration and mucus production. Therefore, it can be considered that CRE has a therapeutic effect on allergic asthma.

The determinant of allergic asthma initiation and severity is associated with an imbalance between oxidants and antioxidants of the airway [3,4]. During the development of allergic asthma, an imbalance between oxidants and antioxidants occurs, and accordingly, levels of antioxidants such as HO-1 and superoxide dismutase (SOD) decrease, whereas levels of oxidants such as ROS and RNS increase substantially [19]. Particularly, Nrf2, acting as an antioxidant protein, moves into the nucleus during the development of asthma after dissociation from Kelch-like ECH-associated protein 1 [20]. This increases the expression of specific proteins, including HO-1, NQO1, and GSH, reducing oxidative damage [20]. Therefore, the increase in the expression of Nrf2 has been reported as an important factor that can suppress the development of allergic asthma through the reduction of oxidative stress [21,22]. Recently, various studies have been reported on therapeutic agents that exert an anti-asthmatic effect by increasing the expression of Nrf2 [16,23]. In this study, we found that CRE increased the translocation of Nrf2 into the nucleus, which eventually elevated the expression of HO-1 and NQO1. These findings indicated that anti-asthmatic effects of CRE are closely related with the elevation of Nrf2 expression.

MMPs are proteolytic enzymes that have the ability to degrade the extracellular matrix and are involved in airway inflammation and remodeling during the development of asthma [24]. In particular, MMP-9 is associated with the occurrence of asthmatic responses by inducing the production of cytokines, chemokines, and growth factors and has been detected in the sputum of patients with allergic asthma [25]. The MMP-9 expression is correlated with phosphorylation of NF-κB, which acts as an important signaling pathway of inflammatory responses [6,26,27]. The increase in NF-κB phosphorylation not only elevates MMP-9 expression but also increases the production of cytokines, which, in turn, exacerbates airway inflammation in the lung tissue during the development of asthma [28]. Additionally, oxidative stress greatly affects the phosphorylation of NF-κB, and when excessive oxidative damage occurs, the phosphorylation of NF-κB is elevated, significantly increasing MMP-9 expression [28]. In this study, we demonstrated that CRE treatment resulted in a reduction in MMP-9 expression with the reduction in phosphorylation of NF-κB in animals with asthma. These findings indicated that the anti-inflammatory effects of CRE were related with the inhibition of NF-κB phosphorylation and MMP-9 expression.

The anti-asthmatic effects of CRE observed in the present study are considered to be related to its active ingredients. Thus, we conducted HPLC analysis to identify its active ingredients. Hydroxycinnamic acid derivatives (HCAs) have been reported as the main bioactive components of the roots of the Cimicifugae species [29]. Quantification of these HCAs is considered to be important in establishing quality control of CRE. Previously, some HPLC analysis methods have been used to assess the quality of Cimicifugae Rhizoma and to analyze HCAs such as caffeic, ferulic, and isoferulic acids [12,30]. Under the HPLC experimental conditions described in this study, five HCAs, namely caffeic acid, cimicifugic acid B, cimicifugic acid F, ferulic acid, and isoferulic acid were identified and quantified in the CRE. These HCAs have various pharmacological properties, including anti-inflammatory and antioxidant properties [31,32,33,34,35]. In particular, caffeic and ferulic acids suppressed allergic responses and airway inflammation in experimental allergic asthma models [31,33].

## 5. Conclusions

In conclusion, we have shown that CRE successfully attenuated inflammatory responses and oxidative stress in asthma induced by OVA. The therapeutic effects of CRE were closely involved with the activation of Nrf2/HO-1/NQO1 signaling and the suppression of MMP-9 expression and NF-κB phosphorylation. Consequently, CRE is thought to be an effective therapeutic agent for allergic asthma.

## Figures and Tables

**Table 1:** Contents of five compounds in Cimicifugae Rhizoma.

Compound	Contents (Mean ± SD, *n* = 3)	
	mg/g	%
Caffeic acid	0.62 ± 0.01	0.06
Ferulic acid	1.35 ± 0.04	0.14
Isoferulic acid	8.09 ± 0.24	0.81
Cimicifugic acid B	1.86 ± 0.03	0.19
Cimicifugic acid F	10.93 ± 0.14	1.09

*n* = 3; three separate samples from Cimicifugae Rhizoma extract (CRE) were weighed and each sample was analyzed once.

## Data Availability

Data are contained within the article or supplementary material.

## Supplementary Materials

The following are available online at https://www.mdpi.com/article/10.3390/antiox10101626/s1, Table S1: Regression equation, linearity, LOD, and LOQ for five compounds (*n* = 3).
