# Supplementary material for: Cimicifugae Rhizoma Extract Attenuates Oxidative Stress and Airway Inflammation via the Upregulation of Nrf2/HO-1/NQO1 and Downregulation of NF-κB Phosphorylation in Ovalbumin-Induced Asthma
Source: Antioxidants (Basel). 2021 Oct 15;10(10):1626. doi: 10.3390/antiox10101626 (PMC8533435; doi:10.3390/antiox10101626)
Supplement: Supplementary file 1 [file antioxidants-10-01626-s001.zip › antioxidants-1392937-supplementary.pdf]

Table S1. Regression equation, linearity, LOD, and LOQ for five compounds (n = 3).

Compound	Regression Equation [a]	Linear Range (μg/mL)	Linearity (R^2)	LOD [b] (μg/mL)	LOQ [c] (μg/mL)
Caffeic acid	y = 24.632x + 2.4567	2–10	0.9995	0.02	0.05
Ferulic acid	y = 27.354x + 11.697	10–50	0.9997	0.10	0.31
Isoferulic acid	y = 26.817x + 34.38	40–200	0.9997	0.36	1.08
Cimicifugic acid B	y = 12.615x + 1.545	10–50	0.9998	0.07	0.22
Cimicifugic acid F	y = 11.459x + 6.525	40–200	0.9997	0.30	0.89

[a] y, peak area of compound; x, concentration (μg/mL) of compound. [b] LOD, limit of detection, S/N = 3. [c] LOQ, limit of quantification, S/N = 10.
